# Occupational injuries in Ethiopia: an umbrella review of prevalence, factors, and prevention from systematic reviews and meta-analyses

**DOI:** 10.1186/s12889-026-27339-8

**Published:** 2026-04-14

**Authors:** Maru Meseret Tadele, Getaye Tizazu, Gizaw Hailiye, Zegeye Regasa, Andualem Fentahun, Desalegn Meseret Tadele

**Affiliations:** Department of Health Informatics, College of Medicine and Health Sciences, Debre Markos University, Debre Markos, Ethiopia

**Keywords:** Occupational injuries, Healthcare workers, Construction workers, Personal protective equipment, Associated factors, Prevention strategies, Umbrella review, Ethiopia

## Abstract

**Background:**

Occupational injury remains a major public health concern globally, contributing significantly to morbidity, mortality, and economic loss. In Ethiopia, several systematic reviews and meta-analyses have examined the burden and determinants of occupational injuries; however, their findings remain scattered, methodologically inconsistent, and spread across multiple fields. So far, no comprehensive synthesis has been undertaken to offer an integrated understanding of the extent and causes of occupational injuries within the Ethiopian context. To address this gap, this umbrella review aimed to synthesize and compare evidence from existing systematic reviews and meta-analyses on the prevalence, associated factors, and prevention strategies of occupational injuries among Ethiopian workers.

**Methods:**

A comprehensive literature search was conducted across six databases—PubMed/MEDLINE, Cochrane Library, EMBASE, CINAHL, Web of Science, and Scopus—to identify eligible systematic reviews and meta-analyses published between 2019 and 2025. Due to high overlap and methodological variability, results were synthesized narratively using tabular and thematic analysis.

**Results:**

Eight eligible systematic reviews and meta-analyses encompassing 50,586 participants were included, representing multiple sectors including healthcare, construction, textile, and waste management. Pooled prevalence of occupational injury ranged from 28.8% to 46.78%, with healthcare workers reporting lower injury rates. Common injury types included falls, lacerations, fractures, needle stick injuries, and workplace violence. Commonly identified associated factors included lack of personal protective equipment, inadequate safety training, and gender. Prevention strategies emphasized personal protective equipment use, safety training, supervision, and zero-tolerance policies for workplace violence.

**Conclusions:**

Occupational injuries in Ethiopia are common, preventable, and unevenly distributed across sectors and regions. Strengthening prevention through consistent PPE use, safety training, and workplace regulation should be prioritized by policymakers and employers.

**Trial registration:**

This study did not involve a clinical trial; therefore, inclusion of a clinical trial registration number is not applicable.

**Supplementary Information:**

The online version contains supplementary material available at 10.1186/s12889-026-27339-8.

## Background

Occupational injury is any injury, illness, or death that arises from an incident that occurs at work [1]. It remains the world’s major public health concern. Global estimates as of 2023 showed that 2.93 million work-related deaths and 395 million non-fatal occupational injuries occurred annually [2]. For low- and middle-income nations like Ethiopia, where labor-intensive industries employ the majority of the workers, the situation is more difficult [3].

Globally, evidence suggests that the burden of occupational injuries extends far beyond individual suffering and loss, encompassing high economic costs that affect individuals, communities, employers, governments, and entire countries [4, 5]. For example, a study examining the economic burden of work-related injuries and diseases in five European Union countries found that these costs ranged from 2.7% to 10.4% of gross domestic product (GDP), depending on the country [4]. According to another study conducted in Ethiopia, the overall economic expenses resulting from occupational injuries in the sugar industry came to 22.6 million Ethiopian Birr [6].

According to Article 95 of the Ethiopian Labour Proclamation No. 1156/2019, occupational injuries include employment accidents that occur during or in connection with work, as well as occupational diseases resulting from exposure to workplace risk factors [7]. Despite variations across industries and injury types, occupational injuries remain a significant public health concern in Ethiopia [8–15]. Occupational injuries among healthcare workers in developing countries were statistically associated with factors such as sex and hours worked, occupational stress, occupation, age, training in infection prevention, use of universal precautions, recapping needles, ward work experience, staffing and resource adequacy, awareness, outdated guidelines, and prior exposure to sharp injury [16]. The following factors were shown to impact occupational injury in Ethiopian institutions other than the healthcare sector: sex [11], working more than eight hours per day [11], PPE use [10, 11, 17], night shift [17], occupational safety training [10, 11, 18], frequent supervision [10, 11], sleep disturbance [18], and education [18].

Between 2019 and 2025, numerous systematic reviews and meta-analyses have been carried out in Ethiopia to explore occupational injuries and the factors influencing them across various sectors. These studies typically target specific groups or industries, such as healthcare professionals [8, 13, 14, 19], construction workers [10, 11, 20], and employees in industrial or factory settings [9, 17, 18, 21, 22]. Despite this growing body of research, the findings remain scattered, methodologically inconsistent, and spread across multiple fields. So far, no comprehensive synthesis has been undertaken to offer an integrated understanding of the extent and causes of occupational injuries within the Ethiopian context.

Because systematic reviews and meta-analyses represent the highest level of the evidence hierarchy [23, 24], having multiple reviews on the same topic—each limited to specific subgroups—creates methodological redundancy and makes sector-wide comparison difficult. This fragmentation reduces their utility for programmatic and policy decision-making. In this context, the importance of a review of systematic reviews—also known as an umbrella review—can play a critical role in synthesizing diverse evidence. The findings of several systematic reviews and meta-analyses can be compiled, summarized, synthesized, and compared. It also integrates research from various disciplines addressing the same topic, helping to identify patterns and produce a high-level, comprehensive synthesis [25, 26]. By consolidating complex and fragmented information into a single, coherent synthesis, this umbrella review would enhance the usability of existing research and support decision makers, planners, researchers, and other stakeholders in using the evidence for making decisions based on evidence [27]. This strategy is particularly critical for resource-limited countries such as Ethiopia to minimize the financial and operational expenses associated with decisions made without evidence.

Therefore, this umbrella review aimed to synthesize the pooled prevalence and associated factors of occupational injuries reported in existing systematic reviews and meta-analyses across different sectors and populations in Ethiopia. It also seeks to compare and summarize key findings to inform planners, policymakers, researchers, and other stakeholders. Furthermore, this review will identify evidence gaps and provide recommendations for future research.

## Methods

### Study design and protocol registration

This study employed an umbrella review of systematic reviews and meta-analyses on occupational injury in Ethiopia. The review protocol was registered in PROSPERO (registration number: CRD420251105324) before the initiation of the study. The protocol detailed the study objectives, eligibility criteria, search strategy, data extraction methods, predictor pooling approach, and quality assessment procedures to ensure transparency and minimize bias. At this stage, the review adhered fully to the protocol, with no deviations from the planned methodology.

### Search strategy

Systematic reviews and meta-analyses were identified through comprehensive literature searches conducted in the following databases: PubMed/MEDLINE, Cochrane Library, EMBASE, CINAHL, Web of Science, and Scopus. Access to subscription-based databases was obtained via an institutional email account. To avoid duplication of effort, the availability of an existing umbrella review protocol was also checked in the PROSPERO database. The literature search was conducted using the following Boolean search string: (“occupational injury” OR “work-related injury” OR “workplace accident” OR “work injury”) AND (“prevalence” OR “magnitude” OR " proportion”) AND (“risk factors” OR “associated factors” OR “predictors” OR “occupational exposure”) AND (“workers” OR “employees” OR “healthcare providers” OR “construction workers” OR “industry workers”) AND “prevention” OR “preventive measures” AND Ethiopia. Filters applied included meta-analysis and systematic review study designs, English language, and publication years from 2019 to 2025 (Supplementary file).

### Eligibility criteria

This review included systematic reviews and meta-analyses that primarily reported on the prevalence, associated factors (or risk factors), prevention strategies, or a combination thereof, related to occupational injury among Ethiopian workers. Eligible studies were published and unpublished but conducted in English since 2019. Reviews that lacked a systematic methodology—such as narrative reviews, scoping reviews, or traditional literature reviews—were excluded. Systematic reviews and meta-analyses that did not include Ethiopian workers in their analyses were also excluded. This review included only studies that defined occupational injury as any injury, illness, or death resulting from an incident or accident occurring in the course of work, as determined through self-report and/or medical confirmation. The inclusion of SRMAs in this review is primarily due to their position at the top of the healthcare evidence hierarchy [23, 24]. Limiting the SRMAs to those published in English reflects a desire to reach a global audience, while the timeframe from 2019 to 2025 was selected to ensure the inclusion of the most recent evidence.

### Selection of studies

Two experts, Maru Meseret and Gizaw Hailiye, independently selected articles using predefined inclusion criteria: the articles had to be systematic reviews or meta-analyses, published between 2019 and 2025 in English, focused on Ethiopian workers, and available in full text.

### Data extraction and synthesis

First, the content and data from each selected article were independently extracted by both experts (Maru Meseret and Gizaw Hailiye). Next, they exchanged articles and repeated the data extraction independently. The level of agreement between the two rounds was evaluated, and no disagreement was observed between the data collectors.

After selecting appropriate articles, data on authors, year of publication, number of primary articles included in the SRMA, sample size, population/setting, pooled prevalence of occupational injury, occupation/industry, region, quality assessment tool used, review type, heterogeneity (I²), sensitivity analysis, databases searched, publication bias assessment (e.g., Egger’s test, funnel plot), GRADE evidence quality, and funding source (if applicable) were retrieved. To determine the appropriate analytical method for this umbrella review, the extent of overlap among primary studies included in the systematic reviews and meta-analyses (SRMAs) was assessed using the Corrected Covered Area (CCA) metric [28]. The cut-off points for determining the level of overlap among primary studies across the included SRMAs were adopted from Kirvalidze et al., where an overlap of 0%–5% was classified as “slight,” 6%–10% as “moderate,” 11%–15% as “high,” and greater than 15% as “very high” overlap [29].

Calculation of the CCA began with the development of a citation matrix, where each row represented a primary study and each column a review; studies were marked “1” if included and “0” if not. The results revealed a slight overall overlap across all SRMAs (CCA = 5.6%). However, pairwise CCA values ranged from 0% to complete overlap, indicating some review pairs shared no studies at all, while others had substantial duplication (Table 1).

**Table 1:** Corrected Covered Area (CCA) analysis indicating overlap among systematic reviews and meta-analyses on occupational injury in Ethiopia, 2025

SerNo	Comparison pair	Times studies appear in review (*N*)	Number of rows (*r*)	Number of reviews (c)	CCA (%)	Decision
	Overall	124	89	8	5.6	Moderate
1	Kaweti G, Feleke T, 2024 [13] Vs. Meseret M. et al., 2021[10]	34	34	2	0.0	Slight
2	Kaweti G, Feleke T, 2024 [13] Vs. Abdulwehab S, Kedir F., 2025 [14]	28	28	2	0.0	Slight
3	Kaweti G, Feleke T, 2024 [13] Vs. Yazie TD. et al., 2019 [8]	47	40	2	17.5	Very high
4	Kaweti G, Feleke T, 2024 [13] Vs. Gietaneh W. et al., 2023 [12]	39	33	2	18.2	Very high
5	Kaweti G, Feleke T, 2024 [13] Vs. Ferede YA. et al., 2025 [15]	31	28	2	10.7	Moderate
6	Kaweti G, Feleke T, 2024 [13] Vs. Alamneh YM. et al., 2020 [9]	45	33	2	36.4	Very high
7	Kaweti G, Feleke T, 2024 [13] Vs. Ashuro Z. et al., 2021 [11]	32	25	2	28.0	Very high
8	Meseret M. et al., 2021[10] Vs. Abdulwehab S, Kedir F., 2025 [14]	18	18	2	0.0	Slight
9	Meseret M. et al., 2021[10] Vs. Yazie TD. et al., 2019 [8]	37	30	2	23.3	Very high
10	Meseret M. et al., 2021[10] Vs. Gietaneh W. et al., 2023 [12]	29	23	2	26.1	Very high
11	Meseret M. et al., 2021[10] Vs. Ferede YA. et al., 2025 [15]	21	18	2	16.7	Very high
12	Meseret M. et al., 2021[10] Vs. Alamneh YM. et al., 2020 [9]	35	33	2	6.1	Moderate
13	Meseret M. et al., 2021[10] Vs. Ashuro Z. et al., 2021 [11]	22	15	2	46.7	Very high
14	Abdulwehab S, Kedir F., 2025 [14] Vs. Yazie TD. et al., 2019 [8]	31	24	2	29.2	Very high
15	Abdulwehab S, Kedir F., 2025 [14] Vs. Gietaneh W. et al., 2023 [12]	23	17	2	35.3	Very high
16	Abdulwehab S, Kedir F., 2025 [14] Vs. Ferede YA. et al., 2025 [15]	15	12	2	25.0	Very high
17	Abdulwehab S, Kedir F., 2025 [14] Vs. Alamneh YM. et al., 2020 [9]	29	17	2	70.6	Very high
18	Abdulwehab S, Kedir F., 2025 [14] Vs. Ashuro Z. et al., 2021 [11]	16	9	2	77.8	Very high
19	Yazie TD. et al., 2019 [8] Vs. Gietaneh W. et al., 2023 [12]	42	29	2	44.8	Very high
20	Yazie TD. et al., 2019 [8] Vs. Ferede YA. et al., 2025 [15]	34	24	2	41.7	Very high
21	Yazie TD. et al., 2019 [8] Vs. Alamneh YM. et al., 2020 [9]	48	29	2	65.5	Very high
22	Yazie TD. et al., 2019 [8] Vs. Ashuro Z. et al., 2021 [11]	35	21	2	66.7	Very high
23	Gietaneh W. et al., 2023 [12] Vs. Ferede YA. et al., 2025 [15]	26	17	2	52.9	Very high
24	Gietaneh W. et al., 2023 [12] Vs. Ferede YA. et al., 2025 [15]	40	22	2	81.8	Very high
25	Gietaneh W. et al., 2023 [12] Vs. Ashuro Z. et al., 2021 [11]	27	14	2	92.9	Very high
26	Ferede YA. et al., 2025 [15] Vs. Alamneh YM. et al., 2020 [9]	32	17	2	88.2	Very high
27	Ferede YA. et al., 2025 [15] Vs. Ashuro Z. et al., 2021 [11]	19	9	2	111.1	Very high
28	Alamneh YM. et al., 2020 [9] Vs. Ashuro Z. et al., 2021 [11]	33	14	2	135.7	Very high

A quantitative pooling (meta-meta-analysis) was considered inappropriate for this umbrella review due to marked methodological heterogeneity between the meta-analyses included [30, 31]. The majority of meta-analyses overlapped in their primary studies, resulting in substantial between-effect size dependency. When coupled with substantial heterogeneity in study populations, interventions, outcome definitions, and analytic approaches, this overlap heightens the risk of biasing aggregated effect estimates and compromising the integrity of a meta-analysis at a higher order. Since these conditions did not meet the assumptions for meta-meta-analysis, we used a narrative approach with tables and thematic methods to provide a transparent, context-sensitive summary of the evidence. Thematic categories were derived from the characteristics of the included studies, the study designs and methods used, the outcomes reported, and the factors identified as influencing these outcomes.

### Quality assessment

Methodological rigor of the included systematic reviews and meta-analyses was assessed using the AMSTAR 2 tool, a validated 16-item checklist [32]. Protocol registered before commencement of the review (item 2), adequacy of the literature search (item 4), justification for excluding individual studies (item 7), risk of bias from individual studies being included in the review (item 9), appropriateness of meta-analysis methods (item 11), consideration of risk of bias when interpreting the results of the review (item 13), and assessment of presence and likely impact of publication bias (item 15) were considered as critical domains. Accordingly, the overall confidence rating for each review was classified as follows: High when there was no or only one non‑critical weakness; Moderate when there were more than one non‑critical weakness but no critical flaw; Low when there was one critical flaw (with or without non‑critical weaknesses); and Critically Low when there were more than one critical flaw [32].

**Fig. 1:**
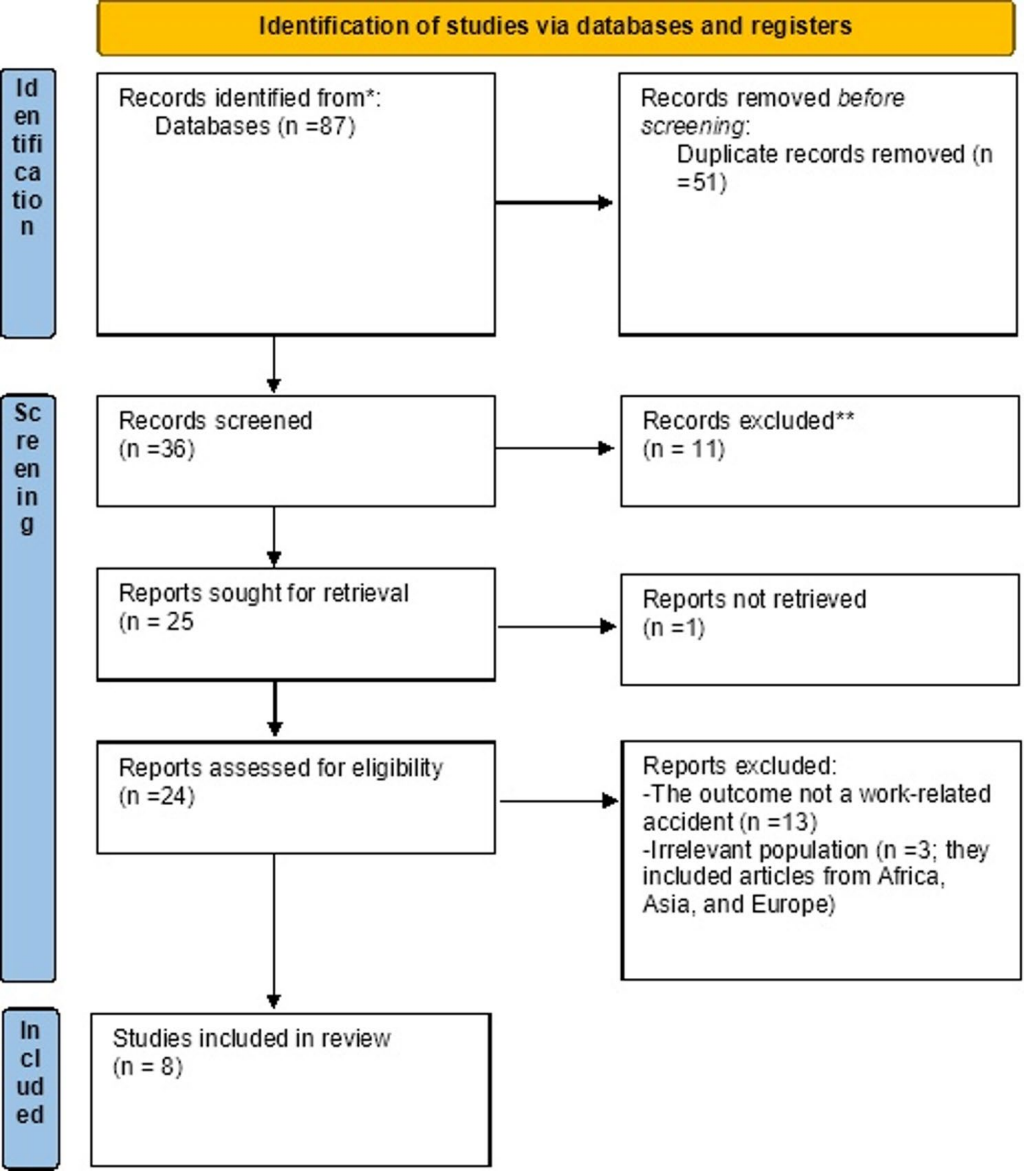
PRISMA 2020 flow diagram for new systematic reviews which included searches of databases and registers only (Adopted from Page MJ, McKenzie JE, Bossuyt PM, Boutron I, Hoffmann TC, Mulrow CD, et al. The PRISMA 2020 statement: an updated guideline for reporting systematic reviews. BMJ. 2021. )

Two independent reviewers (Andualem Fentahun and Desalegn Meseret) applied the tool to evaluate the quality of each eligible article. Although a third reviewer was designated to resolve potential discrepancies, no disagreements were observed between the initial assessors (Table 2). The PRISMA 2020 flowchart [33] was employed as a standardized visual tool to enhance clarity, ensure reproducibility, and foster trust throughout the review process (Fig. 1).

**Table 2:** AMSTAR 2 quality assessment of systematic reviews and meta-analyses on occupational injury in Ethiopia, 2025

Item No	Shortened AMSTAR 2 Question	Response by author with publication year							
		1	2	3	4	5	6	7	8
1	Were PICO elements clearly defined?	Yes	Yes	Yes	Yes	Yes	Yes	Yes	Yes
2	Was a protocol pre-registered, and were deviations justified?	No	No	Yes	Yes	No	Yes	Yes	Yes
3	Was the study design selection clearly explained?	Yes	Yes	Yes	Yes	Yes	Yes	Yes	Yes
4	Was the literature search comprehensive?	Yes	Yes	Yes	Yes	Yes	Yes	Yes	Yes
5	Was study selection done by two reviewers?	Yes	Yes	Yes	Yes	Yes	Yes	Yes	Yes
6	Was data extraction performed in duplicate?	Yes	Yes	Yes	Yes	Yes	Yes	Yes	Yes
7	Were the excluded studies listed and justified?	Par.	Par.	Par.	Par.	Par.	Par.	Par.	Par.
8	Were the included studies described in detail?	Yes	Yes	Yes	Yes	Yes	Yes	Yes	Yes
9	Was the **risk of bias (**RoB) in individual studies appropriately assessed?	Yes	Yes	Yes	Yes	Yes	Yes	Yes	Yes
10	Were funding sources of the included studies reported?	No	No	No	No	No	No	No	No
11	Was a meta-analysis conducted using appropriate methods?	Yes	Yes	Yes	Yes	Yes	Yes	Yes	Yes
12	Was the impact of RoB on synthesis results assessed?	No	No	No	No	No	No	No	No
13	Was RoB considered in interpreting review findings?	Par.	Par.	Par.	Par.	Par.	Par.	Par.	Par.
14	Was heterogeneity explained and discussed?	Yes	Yes	Yes	Yes	Yes	No	Yes	Yes
15	Was publication bias investigated and discussed?	Yes	Yes	Yes	Yes	Yes	Yes	Yes	Yes
16	Were conflicts of interest and funding for the review disclosed?	Yes	Yes	Yes	Yes	Yes	Yes	Yes	Yes
**Critical domains identified**		2,7,13	2,7,13	7, 13	7, 13	2, 7, 13	7, 13	7, 13	7, 13
**Overall confidence rating**: high, moderate, low, critically low (CL)		CL	CL	CL	CL	CL	CL	CL	CL

The numbers 1–8 in the top row above the ‘Yes/No’ responses correspond to the authors and publication years of the included SRMAs, as follows: 1 = Yazie TD et al., 2019 [8]; 2 = Alamneh YM et al., 2020 [9]; 3 = Meseret M et al., 2021; 4 = Ashuro Z et al., 2021 [11]; 5 = Gietaneh W et al., 2023 [12]; 6 = Kaweti G and Feleke T, 2024 [13]; 7 = Abdulwehab S and Kedir F, 2025 [14]; and 8 = Ferede YA et al., 2025 [15], and Par. Stands for partial

### Statistical analysis

Statistical analyses were limited to the assessment of overlap among primary studies using the CCA. No quantitative pooling or meta-meta-analysis was conducted due to substantial methodological heterogeneity and dependency among effect sizes. Data synthesis was therefore narrative, supported by tabular and thematic comparisons of extracted statistical findings.

## Results

### Search result

A total of 87 systematic reviews and meta-analyses were initially retrieved from database searches. Of these, 51 were excluded during the early identification stage due to database duplicates, resulting in 36 unique records. These were further screened by title, abstract, and full-text availability; 11 were excluded based on relevance, and 1 could not be retrieved despite efforts, leaving 24 articles for eligibility assessment. Upon full-text evaluation, 13 articles were excluded for not meeting the definition of occupational injury adopted in this review, while 3 were excluded for incorporating primary studies conducted outside of Ethiopia. Ultimately, 8 systematic reviews and meta-analyses focused exclusively on the Ethiopian context were included (Fig. 1).

### Characteristics of included articles

The eight included SRMAs were based on primary studies ranging from 6 to 25 articles, with participant numbers ranging from 2,826 to 10,996, yielding a total of 50,586 participants included in this review. Seven of the eight SRMAs were based exclusively on primary studies with a cross-sectional design, while the remaining SRMA included 15 primary studies with a cross-sectional design and 2 primary studies with a case-control design. Among the included SRMAs, 4 (50%) focused directly on occupational or work-related injuries, 2 (25%) addressed workplace violence, and the remaining 2 (25%) examined needlestick and sharp object injuries. The included articles were published between 2019 and 2025 (Table 3).

**Table 3:** Characteristics of included systematic reviews and meta-analyses on occupational injury in Ethiopia, 2025

Author & Publication Year	Study region	Design of primary studies	No of primary articles included	Pooled sample size	Pooled prevalence with 95% CI	Quality Assessment Tool Used	Heterogeneity (I²)	Sensitivity Analysis	Publication Bias Assessment
Yazie TD. et al., 2019 [8]	Amhara, Oromia, SNNPR, Somali, Harari, Addis Ababa, Dire Dawa	Cross-sectional	23	7468	28.8% (23.0%–34.5%)	JBI checklist for prevalence studies	97.3% — indicating high heterogeneity	Conducted — no single study significantly influenced pooled estimates	Yes but bias present with *p* = 0.001
Alamneh YM. et al., 2020 [9]	Addis Ababa, Amhara, Oromia, SNNPR, Afar, Gambella, Dire Dawa, Harari	Cross-sectional	23	10,996	44.66% (43.83%–45.49%)	Modified Newcastle-Ottawa Scale for cross-sectional studies	99.2%, indicating substantial heterogeneity	Conducted—4 studies identified as influential and excluded from the final model	Yes, but no significant bias
Meseret M. et al., 2021[10]	Amhara, Addis Ababa, Oromia, SNNPR, Tigray	Cross-sectional	12	5992	45.64% (33.54%–57.74%)	JBI-Meta-Analysis of Statistics Assessment and Review Instrument	99.1%, indicating high heterogeneity	Not reported	Yes, but no significant bias
Ashuro Z. et al., 2021 [11]	Addis Ababa, Oromia, Amhara	Cross-sectional	10	4282	46.78% (32.17%–61.38%)	JBI checklist for prevalence studies	99.0%, indicating substantial heterogeneity	Not reported	Yes but no significant bias
Gietaneh W. et al., 2023 [12]	Addis Ababa, Amhara, Oromia, SNNPR	Cross-sectional (15) & case-control (2)	17	8025	43.59% (34.48%–52.70%)	Modified Newcastle-Ottawa Scale for cross-sectional studies	98.7%, indicating very high heterogeneity	Not reported	Yes but Significant bias
Kaweti G, Feleke T, 2024 [13]	Ethiopian regions (unspecified)	Cross-sectional	22	8087	40.5% (35%–45.9%)	JBI	96.18% (high heterogeneity)	Not reported	Yes but no significant bias
Abdulwehab S, Kedir F., 2025 [14]	Southern, Northwestern, Northeastern, Eastern Ethiopia	Cross-sectional	6	2910	39.61% (36.5%–42.7%)	AXIS tool for cross-sectional studies	58.4%, indicating moderate heterogeneity	Conducted—results ranged from 36.66% to 44.36% upon study exclusion	Yes but no significant bias
Ferede YA. et al., 2025 [15]	Amhara and SNNPR	Cross-sectional	7	2826	39.43% (27.63%–51.23%)	Modified Newcastle-Ottawa Scale for cross-sectional studies	100%, indicating very high heterogeneity	Conducted—no single study significantly influenced pooled estimate	Yes but no significant bias

Of the eight SRMAs, the inclusion of participants from the Amhara region was explicitly described in six articles, followed by Oromia, Addis Ababa, and the Southern Nations, Nationalities, and People’s Region (SNNPR), each represented in five articles. Participants from Tigray, Somali, and Afar were each explicitly described in only one article. One of the SRMAs was pooled from primary studies conducted in Southern, Northwestern, Northeastern, and Eastern Ethiopia, while another was based on studies conducted across various Ethiopian regions (Table 3).

The reviews included workers from several industries in Ethiopia, including the healthcare, construction, textile, and solid waste management sectors. Half of the SRMAs included in this review focused on occupational injury among healthcare workers, two (25%) focused on construction workers, and the remaining two (25%) focused on workers from a variety of industries, including the textile and solid waste management sectors (Table 3).

### Overall quality of included SRMAs

Variable quality appraisal tools were utilized by all included SRMAs, and these tools were used to determine the overall quality of the main studies that were included. The JBI Meta-Analysis of Statistics Assessment and Review Instrument (JBI-MAStARI), the modified Newcastle-Ottawa Scale (NOS) for cross-sectional studies, the JBI checklist for prevalence studies, and the AXIS tool for cross-sectional studies were some of the quality appraisal tools used in the SRMAs. These instruments are used to evaluate the quality and bias risk of observational studies in SRMAs. In particular, the JBI Checklist and AXIS tool evaluate cross-sectional and prevalence studies, guaranteeing methodological rigor. The JBI-MAStARI and the modified NOS facilitate organized quality evaluation and direct trustworthy data synthesis.

All of the included SRMAs reported adherence to the Preferred Reporting Items for Systematic Reviews and Meta-Analyses (PRISMA) guidelines to ensure completeness and transparency of reporting. However, there was an inconsistency in specifying which version of PRISMA—2009 or 2020—was used, which may affect the interpretation of reporting quality across studies.

Based on the AMSTAR 2 tool, which is designed to evaluate the methodological quality of systematic reviews and meta-analyses, all included articles, in one way or another, defined the PICO components, specified the study design and search strategy, employed two independent reviewers for study selection, extracted data in duplicate, provided thorough descriptions of the included studies, assessed the risk of bias in the primary studies, conducted meta-analyses, investigated and discussed publication bias, and clearly disclosed conflicts of interest. However, there was an inconsistency between the protocol registration and the explanation and discussion of heterogeneity. Additionally, the listing and justification of excluded studies, as well as the consideration of risk of bias during the interpretation of review findings, were only partially addressed. Notably, none of the included reviews reported assessing the effect of risk of bias in the synthesis results. Accordingly, all the included 8 systematic reviews and meta-analyses were classified as having critically low confidence because all of them had > 1 critical flaw as per the criteria (Table 3).

Regarding the results of the Corrected Covered Area (CCA) metric, which is used to assess the degree of overlap of primary studies across different systematic reviews and meta-analyses (SRMAs), a total of 28 pairs of reviews were evaluated. Among these, 3 pairs (10.7%) showed slight overlap (e.g., Kaweti vs. Meseret, Kaweti vs. Abdulwehab), and another 3 pairs (10.7%) exhibited moderate overlap (e.g., Meseret vs. Alamneh, Kaweti vs. Ferede). The remaining majority—22 pairs (78.6%)—demonstrated very high overlap, including Alamneh vs. Ashuro (135.7%) and Ferede vs. Ashuro (111.1%) (Table 1). These results indicated that most of the included reviews relied on the same underlying primary studies, and review pairs with a CCA exceeding 100% reflect situations in which one review is essentially a subset of the other. In such cases, the degree of overlap is so substantial that the corrected overlap measure surpasses 100%, demonstrating extreme redundancy in the evidence base.

### Prevalence of occupational injury

**Fig. 2:**
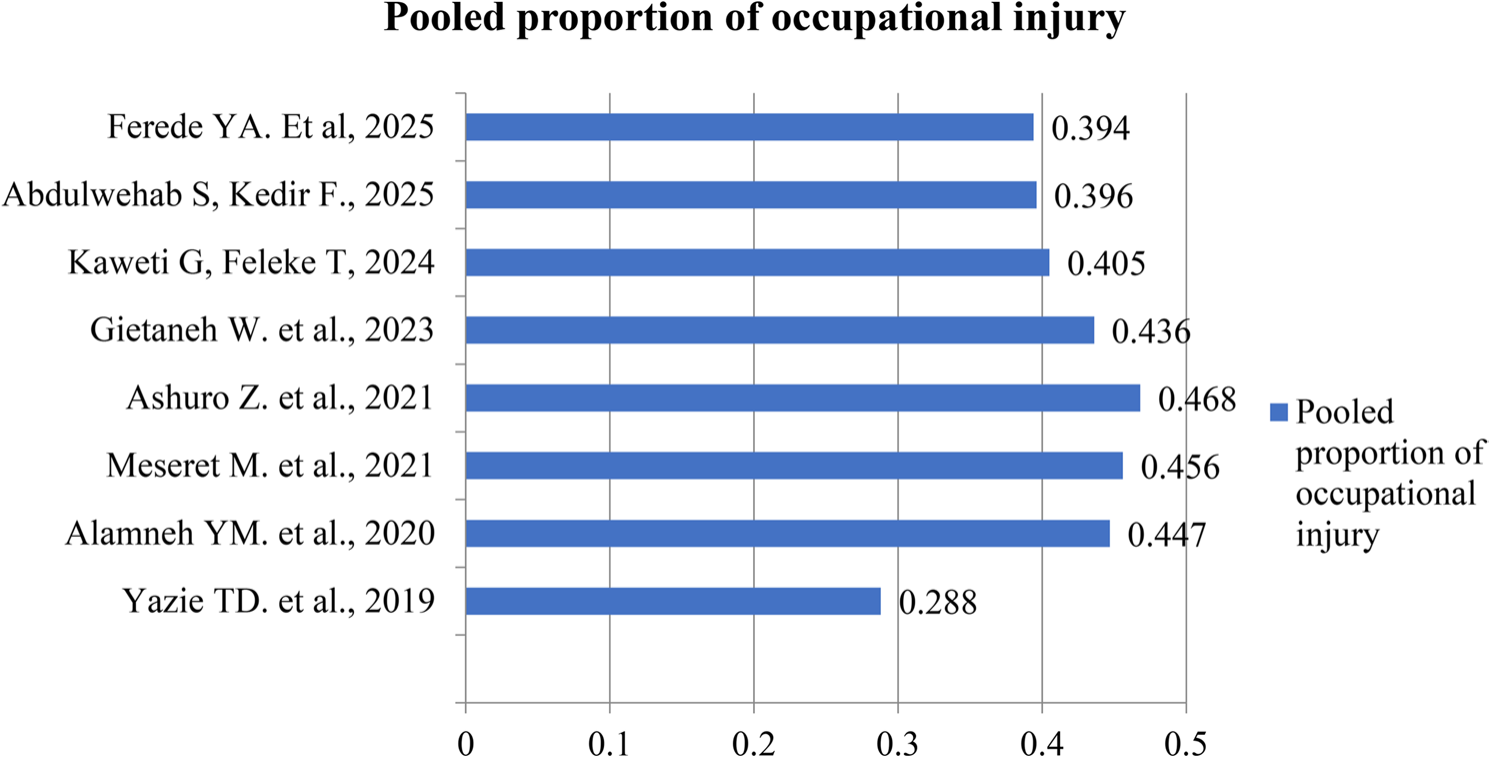
Pooled proportion of occupational injury from included systematic reviews and meta-analyses in Ethiopia, 2025

**Fig. 3:**
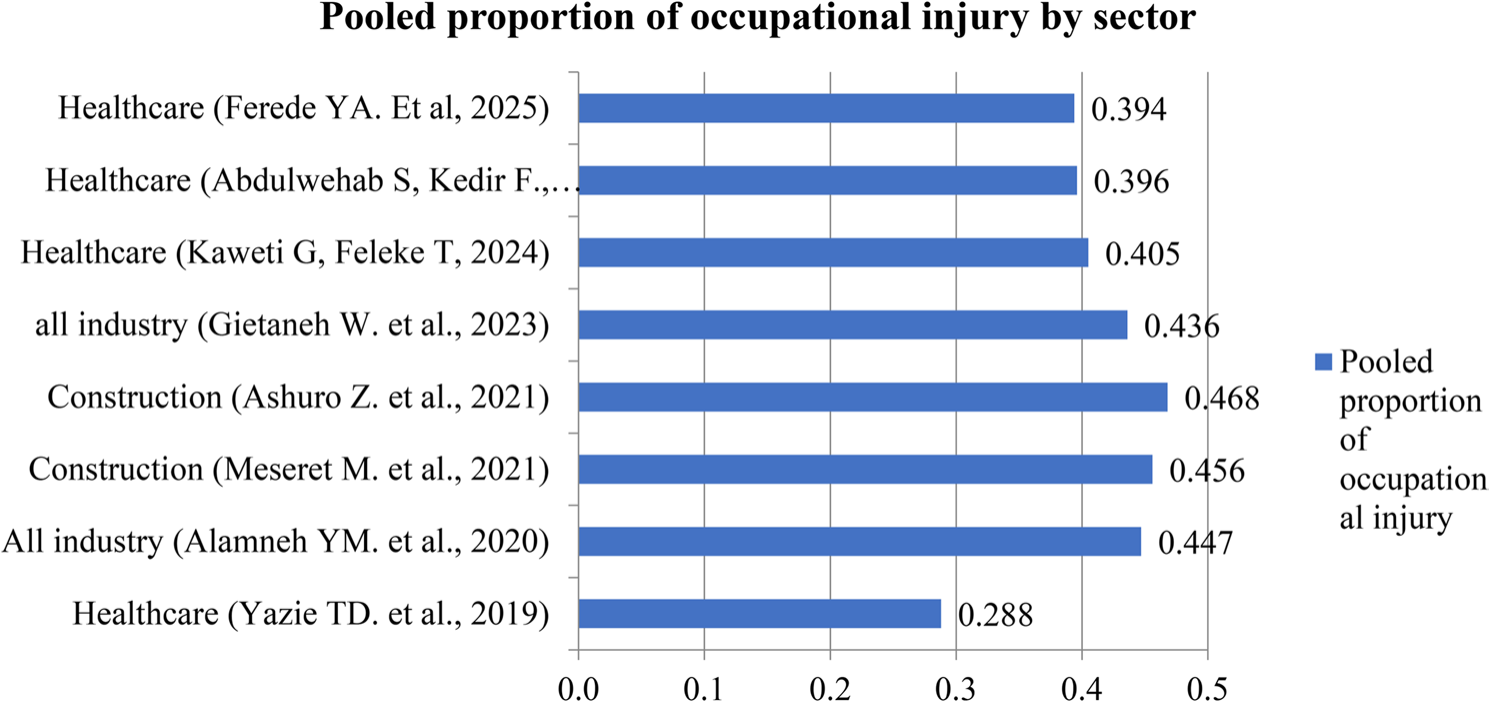
Pooled proportion of occupational injury by sector from included systematic reviews and meta-analyses in Ethiopia, 2025

The pooled prevalence of occupational injury reported in the included systematic reviews and meta-analyses ranged from 28.8% (95% CI: 23.0%–34.5%) [8] to 46.78% (95% CI: 32.17%–61.38%) [11]. Most pooled estimates (62.5%) were concentrated between 40% and 46% (Fig. 2). When stratified by sector, healthcare industries generally reported lower pooled prevalence rates of occupational injury compared to other sectors (Fig. 3).

Among the SRMAs included in the review, occupational injuries among Ethiopian workers—particularly in the construction, healthcare, and industrial sectors—frequently encompass falls from heights, being struck by objects, cuts and lacerations, fractures, burns, electrocution, and musculoskeletal disorders (MSDs). These injuries were primarily attributed to inadequate use of personal protective equipment (PPE), insufficient safety training, and exposure to high-risk environments such as scaffolding, heavy machinery zones, and areas involving sharp tool handling. In healthcare settings, especially among nurses and laboratory personnel, needle stick injuries and blood-borne exposures were recurrent and posed significant occupational hazards. Regarding workplace violence, SRMAs focusing on nursing professionals report high prevalence of verbal abuse, physical assault, bullying, sexual harassment, and psychological intimidation—committed by patients, their relatives, coworkers, and supervisors.

The degree of variability in reported injury rates (I²) across the included studies ranged from 58.4% to 100%. Due to this substantial heterogeneity, all included SRMAs employed a random effects model to pool effect sizes. To identify potential sources of variability, 6 out of 8 SRMAs employed subgroup analyses (Table 4). However, the variables used to define subgroups varied between SRMAs, although some variables—such as region—were used by all SRMAs that did subgroup analysis. It was also identified as a potential source of heterogeneity by all SRMAs that did subgroup analysis. The difference was also observed among SRMAs that used healthcare facility type, sampling technique, study group, type of sector, and study year as a subgroup variable. Sample size was also identified as a source of variability in one of the SRMAs included in this review, given that the other review contradicts the finding.

**Table 4:** Subgroup analysis to identify sources of heterogeneity among included systematic reviews and meta-analyses on occupational injury in Ethiopia, 2025

Authors with publication year	Subgroup variable	Stratification details	Findings
Yazie TD. et al., 2019 [8]	Healthcare facility type	Hospital vs. Hospital & Health Center	Minor variation; heterogeneity remained high
	Sampling technique	Probability vs. Non-probability	Slight difference in prevalence; heterogeneity persisted
	Study group	HCWs vs. HCWs + Cleaners	HCWs alone showed higher prevalence
	Year of publication	2009–2014 vs. 2015–2019	No significant reduction in heterogeneity
	Region	Amhara, Oromia, Others (e.g., Harari, SNNP, Somali, Dire Dawa)	Amhara had lower prevalence; region did not explain heterogeneity fully
Alamneh YM. et al., 2020 [9]	Region	Addis Ababa, Amhara, Oromia, SNNPR	Highest prevalence in Addis Ababa (49.82%), lowest in Oromia (29.55%)
	Occupational site	Construction, Manufacturing, Sewerage/Waste, Health/Social Work	Construction had highest prevalence (50.82%), health sector lowest (29.17%)
	Study year	< 2015 vs. ≥2015	Slight decrease post-MDGs (37.37%) vs. pre-MDGs (45.12%)
Meseret M. et al., 2021[10]	Region	Amhara, Oromia, Addis Ababa, SNNPR, Tigray	Significant variation; region was statistically associated with effect size (*p* = 0.022)
	Sample size	≤ 505 vs. >505 participants	Both strata showed high heterogeneity; not a major source of variation
	Study year	≤ 2015 vs. >2015	Variation observed, but heterogeneity remained high
Ashuro Z. et al., 2021 [11]	Region	Amhara, Oromia, Addis Ababa	Highest prevalence in Addis Ababa (55.9%), lowest in Amhara (35.6%)
	Sample size	< 428 vs. ≥428 participants	Larger samples (≥ 428) showed higher prevalence (52.1%) than smaller ones (43.8%)
Gietaneh W. et al., 2023 [12]	Region	Addis Ababa, Amhara, Oromia, SNNPR	Highest prevalence in Addis Ababa (45.82%), lowest in SNNPR (31.40%)
	Occupation type	MSWM, Construction, Textile	MSWM workers had highest prevalence (45.68%), textile workers lowest (37.44%)
Kaweti G, Feleke T, 2024 [13]	Not reported		
Abdulwehab S, Kedir F., 2025 [14]	Not reported		
Ferede YA. et al., 2025 [15]	Region	Amhara vs. Southern Nations (SNNPR)	Higher prevalence in SNNPR (44.77%) vs. Amhara (35.42%)

A meta-regression using study-level characteristics to explore sources of heterogeneity was conducted in 5 of the 8 included SRMAs (62.5%). The remaining 3 SRMAs did not report performing meta-regression analyses. Region, study year, year of publication, sample size, year of data collection, participant characteristics (not specified in detail), and site of injury were the most commonly used regressors across the included studies. However, only the variable region (*p* = 0.022) was identified by one SRMA as statistically significant, indicating that regional differences accounted for part of the observed variability.

Funnel plot, Egger’s regression test, and Begg’s rank correlation test were the most commonly employed metrics for detecting potential publication bias in systematic reviews with meta-analyses (SRMAs). Accordingly, this umbrella review appraised each included SRMA for the use of these bias detection tools. All SRMAs applied Egger’s regression test, while seven out of eight (87.5%) incorporated a funnel plot. Notably, only two SRMAs (25%)—Gietaneh W. et al., 2023 and Alamneh YM. et al., 2020—employed Begg’s test, reflecting more limited uptake of this method across the evidence base.

Among the included SRMAs, Gietaneh W. et al., 2023 was the only review to explicitly report the presence of publication bias, as evidenced by a statistically significant result in Egger’s regression test (*p* = 0.019). To address this, the authors applied a trim and fill analysis, which estimates and corrects for asymmetry by imputing potentially missing studies, thereby adjusting the pooled effect size to a more conservative estimate.

### Associated factors of occupational injury

A total of seven articles pooled various factors affecting the outcome of interest—occupational injury. The frequency with which these factors were identified varied. Commonly reported associated factors included lack or non-use of personal protective equipment (PPE), lack of safety training, absence of supervision, gender, single marital status, alcohol consumption, substance use (such as khat and smoking), long working hours, assignment to the emergency department, job-related stress, needle recapping, low educational level, short work experience, and night shifts. Among these, lack or non-use of PPE, lack of safety training, and gender were reported in 4 (57.1%) of the included SRMAs, whereas all other factors were mentioned in no more than two studies (Table 5).

**Table 5:** Determinants of occupational injury identified in systematic reviews and meta-analyses in Ethiopia, 2025

SerNo	Author with publication year	Factor	Reference category	AOR with 95%	*P*-value
1	Kaweti G, Feleke T, 2024 [13]	Recapping needles	No (not recapping)	2.3 (1.6, 3.3)	< 0.001
		Absence of routine precautions	No (follow standard precautions	2.3 (1.1, 4.5)	< 0.01
		Lack of training	Yes (had training)	2.4 (1.4, 4.1)	< 0.001
2	Meseret M. et al., 2021[10]	Lack of PPE use	No (used PPE)	1.75 (1.46, 2.10)	< 0.001
		No occupational safety training	No (Had training)	1.63 (1.13, 2.34)	< 0.01
		Absence of regular supervision	No (Had regular supervision)	1.40 (1.16, 1.68)	< 0.001
3	Abdulwehab S, Kedir F., 2025 [14]	Female gender	Male	2.24 (1.07, 3.41)	< 0.01
		Single marital status	Married	4.58 (2.44, 6.73)	< 0.001
		Working night shifts	No	1.57 (1.20, 1.93)	< 0.001
		Emergency department assignment	Not described	3.87 (2.33, 5.41)	< 0.001
		Alcohol consumption	No	2.69 (1.10, 4.28)	< 0.01
4	Yazie TD. et al., 2019 [8]	Not pooled			
5	Gietaneh W. et al., 2023 [12]	Alcohol consumption	No	1.75 (1.03, 2.99)	< 0.05
		Cigarette smoking	No	2.15 (1.32, 3.50)	< 0.01
		Khat chewing	No	2.03 (1.44, 2.86)	< 0.001
		Job-related stress	No	3.85 (2.66, 5.59)	< 0.001
		Education level	Low educational level	0.51 (0.30, 0.89)	< 0.05
		Use of PPE	Yes	0.48 (0.36, 0.65)	< 0.001
6	Ferede YA. et al., 2025 [15]	Female gender	Male	2.25 (1.29, 3.92)	< 0.01
		Short work experience	Long work experience	3.25 (2.37, 4.45)	< 0.001
		Single marital status	Married	2.03 (1.03, 3.99)	< 0.05
7	Alamneh YM. et al., 2020 [9]	Male gender	Female	1.46 (1.01, 2.11)	< 0.05
		Working > 8 h/day	≤ 8 h/day	2.84 (1.81, 4.46)	< 0.001
		Lack of PPE use	No (used PPE)	3.01 (1.61, 5.63)	< 0.001
		Absence of supervision	No (regular supervision)	2.83 (1.58, 5.15)	< 0.001
		No occupational safety training	No (had training)	2.18 (1.40, 3.39)	< 0.001
8	Ashuro Z. et al., 2021 [11]	Lack of safety training	No (had training)	2.43 (1.76, 3.35)	< 0.001
		Not using PPE	No (used PPE)	2.32 (1.80, 2.99)	< 0.001
		Male gender	Female	2.44 (1.15, 5.17)	< 0.05

Workers who were male, recapped needles, were unable to follow standard precautions, lacked occupational safety training, worked more than eight hours a day, did not use personal protective equipment (PPE), and were not regularly supervised had much higher odds of suffering an occupational injury. Workers who engaged in behaviors including drinking alcohol, smoking cigarettes, chewing khat, or experiencing stress at work were more likely to have an occupational injury. Nurses who were female, single, assigned to the emergency department, worked night shifts, drank alcohol, and had little work experience were much more likely to suffer workplace violence. All of these determinants had odds ratios > 1 with 95% confidence intervals, indicating that they were positively associated with higher injury risk (Table 5).

However, workers who used PPE and those with higher educational levels were associated with lower odds of occupational injury (95% confidence interval and odds ratios < 1). An odds ratio < 1 shows a protective effect, meaning these factors reduced the likelihood of injury compared to their reference groups (Table 5).

Sensitivity analyses in the included SRMAs were conducted to assess the presence of potentially skewed estimates that could have inflated the pooled effect size, potentially leading to a misleading or biased overall estimate. This umbrella review evaluated whether sensitivity analyses were performed in the SRMAs. Accordingly, only half (4 out of 8) conducted sensitivity analyses, and among these, two identified influential individual studies. Excluding those studies mitigated the observed issues, suggesting their significant impact on the pooled results (Table 3).

### Prevention strategies

Inadequate personal protective equipment and safety tools, low levels of safety training and awareness, weak supervision and follow-up, gender and experience disparities, as well as psychosocial risks and workplace violence, were the most commonly identified contributing factors to gross occupational injuries. More specifically, systematic reviews and meta-analyses (SRMAs) focusing on workplace violence—classified under occupational injuries—recommended several key preventive strategies. These included creating a safer and more supportive environment for nurses, enforcing zero-tolerance policies against violence, improving patient-nurse relationships, and providing psychological support. These interventions aim to reduce the prevalence of workplace violence, ensure the well-being of healthcare professionals, and emphasize the importance of prioritizing violence prevention efforts.

Additionally, SRMAs focusing on needlestick and sharp object injuries—another major category of occupational injuries—highlighted the importance of implementing workplace safety standards within health facilities. Other critical recommendations included conducting regular follow-ups and providing both on-the-job and in-service training programs.

### Funding

Of all included SRMAs, only 5 (62.5%) disclosed that they did not receive any funding, while the remaining SRMAs did not mention their funding status.

## Discussion

The magnitude, associated factors, and preventive strategies of occupational injury in Ethiopia have not been consistent across primary studies. Furthermore, findings from systematic reviews and meta-analyses have also shown inconsistencies. These conflicting results across SRMAs on the same topic pose challenges for stakeholders, including planners, programmers, scholars, decision-makers, and policymakers. In such situations, umbrella reviews play a crucial role worldwide by synthesizing evidence from multiple reviews [25–27].

Regional disparities in the included SRMAs were apparent. Some regions, such as Afar, Tigray, and Somali, were represented only once, and for at least one region, data on occupational injury were unavailable. These regional disparities highlight substantial gaps in the existing evidence base, underscoring the need for nationally representative research that provides a comprehensive picture of occupational injury in Ethiopia.

Among the 120 primary studies included across the eight SRMAs, 118 (98.3%) utilized cross-sectional designs. Consequently, seven of the eight SRMAs relied exclusively on cross-sectional evidence, constraining the ability to draw causal inferences and introducing potential temporal ambiguity. Moreover, the number of included studies per SRMA varied substantially (range: 6 to 23), with publication years spanning 2019–2025. These discrepancies likely reflect heterogeneity in eligibility criteria, outcome definitions, search cut-off dates, methodological rigor, risk-of-bias thresholds, and search strategy comprehensiveness.

The SRMAs included in the umbrella review used quality assessment tools. However, there was noticeable variation in the tools selected: four SRMAs (50%) used the JBI tools, three (35.5%) applied modified versions of the Newcastle-Ottawa Scale, and the remaining study used the AXIS tool. This variation points to a lack of consistency in how quality was assessed. Several factors may help explain this, including researchers’ familiarity with certain tools, the specific requirements of target journals, the scope and focus of each review, the need to adapt tools to suit the purpose, the balance between thoroughness and ease of use, and how well the tools fit the research context. These differences in quality appraisal tools may limit the comparability of findings across SRMAs, as variations in assessment criteria and scoring could lead to inconsistent evaluations of study quality.

There is an inconsistency in the pooled prevalence of occupational injury among workers, ranging from 28.8% [8] to 46.78% [11], while the pooled prevalence reported in most SRMAs was clustered between 40% and 46%. This range is lower than the estimates reported in Tanzania (65.1%) [34], Ghana (66.7%) [35], and globally (55%) [36]. Heterogeneity in the definition of occupational injury, along with variations in inclusion and exclusion criteria, search strategies, and review periods, may account for the differences observed in the pooled prevalence in the Ethiopian context. The discrepancy between these findings and global estimates could be attributed to differences in study design, study settings, study periods, industry types, and other contextual factors.

Heterogeneity was reduced slightly by subgroup variables such as region, healthcare facility type, sampling technique, study group, type of sector, and study year. This result was supported by global research findings, although those analyses were conducted at a broader scale, considering continents as regions and nations as countries [37, 38]. The reduction due to region could be explained by differences in the type and size of industries between regions, while the reduction of variability between healthcare facilities would also be attributed to differences in protocol, resources, and patient demographics. Moreover, selection bias that would be introduced due to differences in sampling techniques (random vs. convenience) could also be a justification for the reduction of heterogeneity. Over time, sectors can change the funding, training, protocols, interventions, and workload in their industry, which could bring changes in the prevalence of occupational injury.

Differences in occupational health and safety rules, access to resources, and economic development would be ideal reasons for variability in occupational injury by region. Similarly, the type of healthcare facility determines workload, specialization, resources, and protocols, which directly affect occupational injury. Smaller samples would fail to capture the true variability in occupational injuries compared to larger samples. Demographics, occupation-specific risks, experience, and training, which could be considered as study groups, would also have effects on the magnitude of occupational injury. Sectoral culture, including attitudes toward safety, work conditions, and exposure to hazards, contributes to the variability of occupational injuries across different sectors. Policy changes over time, epidemics or crises, and trends in reporting could also bring variability in the magnitude of occupational injury among workers.

The most often cited factors influencing occupational injury—reported in four (57.1%) of the studies reviewed—were gender, lack of or failure to use personal protective equipment (PPE), and inadequate safety training. A similar systematic review and meta-analysis conducted globally also identified failure to use PPE as a significant contributor to occupational injury. This finding supports Ethiopia’s Occupational Safety and Health and Working Environment Policy that obligates employers not only to provide PPE but also to train workers in its proper use. The policy further requires workers to correctly use safety devices and PPE, as stipulated in Labour Proclamation No. 1156/2019, Articles 92 (employer obligations) and 93 (worker obligations) [39]. Taken together, these findings indicate that PPE use, safety training, and gender-related factors were consistently associated with differences in the prevalence of occupational injury.

### Public health and policy implications

The need for targeted prevention strategies—including stronger enforcement of PPE use, routine safety training, and gender-sensitive interventions—has important public health and policy implications. Effective and consistent use of PPE can substantially reduce the burden of occupational injuries, associated healthcare costs, and loss of productivity among industry workers. Similar benefits can be achieved by strengthening workers’ capacities through standardized safety training and by addressing gender-specific risks through interventions that improve knowledge, skills, and awareness while accounting for the unique vulnerabilities of different groups of workers.

### Strengths and limitations

Key strengths of this umbrella review included a comprehensive search for relevant articles across multiple databases, protocol registration in PROSPERO, and assessment of methodological quality using AMSTAR 2. However, several limitations should be noted. One limitation of this review is that only articles published in English were included, which may introduce language bias. The other limitation is the inability to include SRMAs evaluating interventional studies, limiting causal inference. As a result, the review is limited to observational and prevalence data, which restricts the ability to draw causal inferences regarding the effectiveness of preventive measures such as PPE use, safety training, or engineering controls. High and often unexplained heterogeneity was common across reviews, and substantial overlap of primary studies—frequently reaching very high CCA values—prevented meta-meta-analysis and increased the risk of double-counting evidence. In addition, several Ethiopian regions were underrepresented or absent from the underlying evidence base, limiting the national representativeness of the findings.

## Conclusions

In Ethiopia, occupational injuries are prevalent, avoidable, and unequally spread among industries and locations. Although current SRMAs offer insightful information, methodological variability, high study overlap, and regional variation underline the need for stronger, sector-specific, inclusive, and nationally representative research. Ensuring methodological rigor requires the development, adaptation, or adoption of standardized definitions, uniform measurement tools, and comparable data-collection procedures across regions and sectors.

To address the wide variability in reported injury prevalence, future research should incorporate nationally representative designs—ideally, observational studies that capture the what, where, when, and who of occupational injuries. This is especially important for underrepresented regions such as Afar, Tigray, Somali, and untouched regions where SRMAs are scarce or absent. Future research should also focus on gender-specific interventions such as capacity building, PPE utilization, and workplace regulation. To lower injury rates and foster safer work conditions, policymakers and businesses should give first priority to consistent preventative measures, particularly PPE utilization, safety training, and workplace regulation.

## Supplementary Information

Supplementary Material 1

Supplementary Material 2

Supplementary Material 3

## Data Availability

The datasets used and/or analyzed during the current review are available from the corresponding author on reasonable request.

## Abbreviations

AMSTAR 2: A Measurement Tool to Assess Systematic Reviews 2
AXIS: Appraisal Tool for Cross-Sectional Studies
CCA: Corrected Covered Area
I^2^: I-squared statistic (measure of heterogeneity in meta-analysis)
JBI: Joanna Briggs Institute
MSDs: Musculoskeletal Disorders
NOS: Newcastle-Ottawa Scale
PPE: Personal Protective Equipment
PRISMA: Preferred Reporting Items for Systematic Reviews and Meta-Analyses
SNNPR: Southern Nations, Nationalities, and People’s Region
SRMA: Systematic Review and Meta-Analysis
