# Supplementary material for: Occupational injuries in Ethiopia: an umbrella review of prevalence, factors, and prevention from systematic reviews and meta-analyses
Source: BMC Public Health. 2026 Apr 14;26:1674. doi: 10.1186/s12889-026-27339-8 (PMC13195873; doi:10.1186/s12889-026-27339-8)
Supplement: Supplementary file 3 — Supplementary Material 3 [file 12889_2026_27339_MOESM3_ESM.docx]

**Supplementary: Sample search strategy in the PubMed database**

**((((((((((((((((((Occupational injury) OR (work-related injury)) OR (workplace accident)) OR (work injury)) AND (Prevalence)) OR (Magnitude)) OR (Proportion)) AND (risk factors)) OR (associated factors)) OR (predictors)) OR (occupational exposure)) AND (workers)) OR (employees)) OR (healthcare providers)) OR (construction workers)) OR (industry workers)) AND (Prevention)) OR (Preventive measures)) AND (Ethiopia) Filters: Free full text, Meta-Analysis, Systematic Review, English, from 2019 - 2026**

(((((((((("occupational injuries"[MeSH Terms] OR ("occupational"[All Fields] AND "injuries"[All Fields]) OR "occupational injuries"[All Fields] OR ("occupational"[All Fields] AND "injury"[All Fields]) OR "occupational injury"[All Fields] OR ("work-related"[All Fields] AND ("injurie"[All Fields] OR "injuried"[All Fields] OR "injuries"[MeSH Subheading] OR "injuries"[All Fields] OR "wounds and injuries"[MeSH Terms] OR ("wounds"[All Fields] AND "injuries"[All Fields]) OR "wounds and injuries"[All Fields] OR "injurious"[All Fields] OR "injury s"[All Fields] OR "injuryed"[All Fields] OR "injurys"[All Fields] OR "injury"[All Fields])) OR ("accidents, occupational"[MeSH Terms] OR ("accidents"[All Fields] AND "occupational"[All Fields]) OR "occupational accidents"[All Fields] OR ("workplace"[All Fields] AND "accident"[All Fields]) OR "workplace accident"[All Fields]) OR (("work"[MeSH Terms] OR "work"[All Fields]) AND ("injurie"[All Fields] OR "injuried"[All Fields] OR "injuries"[MeSH Subheading] OR "injuries"[All Fields] OR "wounds and injuries"[MeSH Terms] OR ("wounds"[All Fields] AND "injuries"[All Fields]) OR "wounds and injuries"[All Fields] OR "injurious"[All Fields] OR "injury s"[All Fields] OR "injuryed"[All Fields] OR "injurys"[All Fields] OR "injury"[All Fields]))) AND ("epidemiology"[MeSH Subheading] OR "epidemiology"[All Fields] OR "prevalence"[All Fields] OR "prevalence"[MeSH Terms] OR "prevalance"[All Fields] OR "prevalences"[All Fields] OR "prevalence s"[All Fields] OR "prevalent"[All Fields] OR "prevalently"[All Fields] OR "prevalents"[All Fields])) OR ("magnitude"[All Fields] OR "magnitudes"[All Fields]) OR ("proportion"[All Fields] OR "proportions"[All Fields])) AND ("risk factors"[MeSH Terms] OR ("risk"[All Fields] AND "factors"[All Fields]) OR "risk factors"[All Fields])) OR (("associate"[All Fields] OR "associated"[All Fields] OR "associates"[All Fields] OR "associating"[All Fields] OR "association"[MeSH Terms] OR "association"[All Fields] OR "associations"[All Fields]) AND ("factor"[All Fields] OR "factor s"[All Fields] OR "factors"[All Fields])) OR ("predictor"[All Fields] OR "predictors"[All Fields]) OR ("occupational exposure"[MeSH Terms] OR ("occupational"[All Fields] AND "exposure"[All Fields]) OR "occupational exposure"[All Fields])) AND ("occupational groups"[MeSH Terms] OR ("occupational"[All Fields] AND "groups"[All Fields]) OR "occupational groups"[All Fields] OR "worker"[All Fields] OR "workers"[All Fields] OR "worker s"[All Fields])) OR ("employee s"[All Fields] OR "occupational groups"[MeSH Terms] OR ("occupational"[All Fields] AND "groups"[All Fields]) OR "occupational groups"[All Fields] OR "employee"[All Fields] OR "employees"[All Fields]) OR ("health personnel"[MeSH Terms] OR ("health"[All Fields] AND "personnel"[All Fields]) OR "health personnel"[All Fields] OR ("healthcare"[All Fields] AND "providers"[All Fields]) OR "healthcare providers"[All Fields]) OR (("construct s"[All Fields] OR "constructed"[All Fields] OR "constructing"[All Fields] OR "construction"[All Fields] OR "constructions"[All Fields] OR "constructive"[All Fields] OR "constructively"[All Fields] OR "constructs"[All Fields] OR "dna recombinant"[Supplementary Concept] OR "dna recombinant"[All Fields] OR "construct"[All Fields] OR "dna, recombinant"[MeSH Terms] OR ("dna"[All Fields] AND "recombinant"[All Fields]) OR "recombinant dna"[All Fields]) AND ("occupational groups"[MeSH Terms] OR ("occupational"[All Fields] AND "groups"[All Fields]) OR "occupational groups"[All Fields] OR "worker"[All Fields] OR "workers"[All Fields] OR "worker s"[All Fields])) OR (("industrial development"[MeSH Terms] OR ("industrial"[All Fields] AND "development"[All Fields]) OR "industrial development"[All Fields] OR "industrialization"[All Fields] OR "industrialize"[All Fields] OR "industrialized"[All Fields] OR "industrializing"[All Fields] OR "industrially"[All Fields] OR "industrials"[All Fields] OR "industry"[MeSH Terms] OR "industry"[All Fields] OR "industrial"[All Fields] OR "industries"[All Fields] OR "industry s"[All Fields]) AND ("occupational groups"[MeSH Terms] OR ("occupational"[All Fields] AND "groups"[All Fields]) OR "occupational groups"[All Fields] OR "worker"[All Fields] OR "workers"[All Fields] OR "worker s"[All Fields]))) AND ("prevent"[All Fields] OR "preventability"[All Fields] OR "preventable"[All Fields] OR "preventative"[All Fields] OR "preventatively"[All Fields] OR "preventatives"[All Fields] OR "prevented"[All Fields] OR "preventing"[All Fields] OR "prevention and control"[MeSH Subheading] OR ("prevention"[All Fields] AND "control"[All Fields]) OR "prevention and control"[All Fields] OR "prevention"[All Fields] OR "prevention s"[All Fields] OR "preventions"[All Fields] OR "preventive"[All Fields] OR "preventively"[All Fields] OR "preventives"[All Fields] OR "prevents"[All Fields])) OR ("prevention and control"[MeSH Subheading] OR ("prevention"[All Fields] AND "control"[All Fields]) OR "prevention and control"[All Fields] OR ("preventive"[All Fields] AND "measures"[All Fields]) OR "preventive measures"[All Fields])) AND ("ethiopia"[MeSH Terms] OR "ethiopia"[All Fields] OR "ethiopia s"[All Fields])) AND ((ffrft[Filter]) AND (meta-analysis[Filter] OR systematicreview[Filter]) AND (english[Filter]) AND (2019:2026[pdat]))

Translations

Occupational injury: "occupational injuries"[MeSH Terms] OR ("occupational"[All Fields] AND "injuries"[All Fields]) OR "occupational injuries"[All Fields] OR ("occupational"[All Fields] AND "injury"[All Fields]) OR "occupational injury"[All Fields]

injury: "injurie"[All Fields] OR "injuried"[All Fields] OR "injuries"[Subheading] OR "injuries"[All Fields] OR "wounds and injuries"[MeSH Terms] OR ("wounds"[All Fields] AND "injuries"[All Fields]) OR "wounds and injuries"[All Fields] OR "injurious"[All Fields] OR "injury's"[All Fields] OR "injuryed"[All Fields] OR "injurys"[All Fields] OR "injury"[All Fields]

workplace accident: "accidents, occupational"[MeSH Terms] OR ("accidents"[All Fields] AND "occupational"[All Fields]) OR "occupational accidents"[All Fields] OR ("workplace"[All Fields] AND "accident"[All Fields]) OR "workplace accident"[All Fields]

work: "work"[MeSH Terms] OR "work"[All Fields]

injury: "injurie"[All Fields] OR "injuried"[All Fields] OR "injuries"[Subheading] OR "injuries"[All Fields] OR "wounds and injuries"[MeSH Terms] OR ("wounds"[All Fields] AND "injuries"[All Fields]) OR "wounds and injuries"[All Fields] OR "injurious"[All Fields] OR "injury's"[All Fields] OR "injuryed"[All Fields] OR "injurys"[All Fields] OR "injury"[All Fields]

Prevalence: "epidemiology"[Subheading] OR "epidemiology"[All Fields] OR "prevalence"[All Fields] OR "prevalence"[MeSH Terms] OR "prevalance"[All Fields] OR "prevalences"[All Fields] OR "prevalence's"[All Fields] OR "prevalent"[All Fields] OR "prevalently"[All Fields] OR "prevalents"[All Fields]

Magnitude: "magnitude"[All Fields] OR "magnitudes"[All Fields]

Proportion: "proportion"[All Fields] OR "proportions"[All Fields]

risk factors: "risk factors"[MeSH Terms] OR ("risk"[All Fields] AND "factors"[All Fields]) OR "risk factors"[All Fields]

associated: "associate"[All Fields] OR "associated"[All Fields] OR "associates"[All Fields] OR "associating"[All Fields] OR "association"[MeSH Terms] OR "association"[All Fields] OR "associations"[All Fields]

factors: "factor"[All Fields] OR "factor's"[All Fields] OR "factors"[All Fields]

predictors: "predictor"[All Fields] OR "predictors"[All Fields]

occupational exposure: "occupational exposure"[MeSH Terms] OR ("occupational"[All Fields] AND "exposure"[All Fields]) OR "occupational exposure"[All Fields]

workers: "occupational groups"[MeSH Terms] OR ("occupational"[All Fields] AND "groups"[All Fields]) OR "occupational groups"[All Fields] OR "worker"[All Fields] OR "workers"[All Fields] OR "worker's"[All Fields]

employees: "employee's"[All Fields] OR "occupational groups"[MeSH Terms] OR ("occupational"[All Fields] AND "groups"[All Fields]) OR "occupational groups"[All Fields] OR "employee"[All Fields] OR "employees"[All Fields]

healthcare providers: "health personnel"[MeSH Terms] OR ("health"[All Fields] AND "personnel"[All Fields]) OR "health personnel"[All Fields] OR ("healthcare"[All Fields] AND "providers"[All Fields]) OR "healthcare providers"[All Fields]

construction: "construct's"[All Fields] OR "constructed"[All Fields] OR "constructing"[All Fields] OR "construction"[All Fields] OR "construction's"[All Fields] OR "constructions"[All Fields] OR "constructive"[All Fields] OR "constructively"[All Fields] OR "constructs"[All Fields] OR "dna, recombinant"[Supplementary Concept] OR "dna, recombinant"[All Fields] OR "construct"[All Fields] OR "dna, recombinant"[MeSH Terms] OR ("dna"[All Fields] AND "recombinant"[All Fields]) OR "recombinant dna"[All Fields]

workers: "occupational groups"[MeSH Terms] OR ("occupational"[All Fields] AND "groups"[All Fields]) OR "occupational groups"[All Fields] OR "worker"[All Fields] OR "workers"[All Fields] OR "worker's"[All Fields]

industry: "industrial development"[MeSH Terms] OR ("industrial"[All Fields] AND "development"[All Fields]) OR "industrial development"[All Fields] OR "industrialization"[All Fields] OR "industrialize"[All Fields] OR "industrialized"[All Fields] OR "industrializing"[All Fields] OR "industrially"[All Fields] OR "industrials"[All Fields] OR "industry"[MeSH Terms] OR "industry"[All Fields] OR "industrial"[All Fields] OR "industries"[All Fields] OR "industry's"[All Fields]

workers: "occupational groups"[MeSH Terms] OR ("occupational"[All Fields] AND "groups"[All Fields]) OR "occupational groups"[All Fields] OR "worker"[All Fields] OR "workers"[All Fields] OR "worker's"[All Fields]

Prevention: "prevent"[All Fields] OR "preventability"[All Fields] OR "preventable"[All Fields] OR "preventative"[All Fields] OR "preventatively"[All Fields] OR "preventatives"[All Fields] OR "prevented"[All Fields] OR "preventing"[All Fields] OR "prevention and control"[Subheading] OR ("prevention"[All Fields] AND "control"[All Fields]) OR "prevention and control"[All Fields] OR "prevention"[All Fields] OR "prevention's"[All Fields] OR "preventions"[All Fields] OR "preventive"[All Fields] OR "preventively"[All Fields] OR "preventives"[All Fields] OR "prevents"[All Fields]

Preventive measures: "prevention and control"[Subheading] OR ("prevention"[All Fields] AND "control"[All Fields]) OR "prevention and control"[All Fields] OR ("preventive"[All Fields] AND "measures"[All Fields]) OR "preventive measures"[All Fields]

Ethiopia: "ethiopia"[MeSH Terms] OR "ethiopia"[All Fields] OR "ethiopia's"[All Fields]
